# Predictive value of current nodal staging systems and development of machine learning nomogram for resectable pancreatic head cancer: a population-based study and multicenter validation

**DOI:** 10.3389/fimmu.2025.1639186

**Published:** 2025-12-02

**Authors:** Wen-Bo Zou, Xiu-Ping Zhang, Yu-Yao Song, Shuai Xu, Bo-Yuan Liu, Zhi-Ming Zhao, Yuan-Xing Gao, Ming-Gen Hu, Gao Huang, Rong Liu

**Affiliations:** 1 Faculty of Hepato-Biliary-Pancreatic Surgery, The First Medical Center of Chinese People’s Liberation Army (PLA) General Hospital, Beijing, China; 2 Department of General Surgery, Department of Pathology, Clinical Research Center, No.924 Hospital of PLA Joint Logistic Support Force, Guilin, China; 3 Department of Liver Transplantation and Hepatobiliary Surgery, Shandong Provincial Hospital Affiliated to Shandong First Medical University, Jinan, China

**Keywords:** pancreatic head cancer, nodal staging systems, machine learning, overall survival, predictive model

## Abstract

**Background:**

Given the growing interest in the influence of lymph node metastasis on the prognosis of patients diagnosed with pancreatic head cancer (PHC). This study aims to evaluate the ability of current four nodal staging systems predicting long-term outcomes and develop a machine learning model for predicting the prognosis of patients with resectable PHC.

**Materials and methods:**

Participants with PHC were sourced from the Surveillance, Epidemiology, and End Results (SEER) database and allocated at random in a 7:3 ratio to training and internal validation cohort. External validation in a large-sample, multicenter cohort collected from three Chinese institutions was performed to verified the robustness of the optimal nodal staging system and predictive model. The concordance index (C-index), Akaike information criterion (AIC) and area under the curve (AUC) were calculated to evaluate the predictive capability and discrimination of different nodal staging systems. The machine learning procedures based procedure and Cox regression analysis were implemented for identification of the prognostic factors and construction of predictive model. The calibration curves, net reclassification improvement (NRI) and integrated discrimination improvement (IDI) and decision curve analysis (DCA) were using to assess predictive accuracy and clinical benefits of the predictive model.

**Results:**

All four nodal staging systems were independent prognostic factors for overall survival (OS). The log odds of lymph node ratio (LODDS) were verified as the optimal nodal staging system with highest C-index and AUCs, and lowest AICs compared to others, and has better predictive capability than others both in patients with < 12 and ≥ 12 retrieval lymph nodes (RLNs). Then, a predictive model including T stage, tumor differentiation, chemotherapy, and LODDS was developed and validated. This model had a higher C-index and AUCs than the AJCC staging system. The NRI, IDI, and DCA analysis also indicated that present model had good predictive capability and clinical utility.

**Conclusion:**

The nodal staging system LODDS is the optimal prognostic factor for OS in resectable PHC. It could effectively predict OS for resectable PHC patients without considering the numbers of RLN. The machine learning model could effectively predict OS for patients with resectable PHC.

## 1 Introduction

Pancreatic cancer is emerging as a formidable adversary to human health (1, 2). Its insidious nature, coupled with the formidable challenges on early detection and effective treatment, has led to a substantial and growing burden on societies worldwide (3, 4). Although radical resection and adjuvant therapy provide the treatment options, the five-year survival rate for pancreatic cancer is relatively low compared to other gastrointestinal cancers, particularly when diagnosed at a late stage (5). The location of the primary tumor is a critical factor in determining treatment strategies and influencing outcomes. Studies have reported that pancreatic head cancer (PHC) account for approximate 75% of pancreatic cancer, and has a notably poor overall survival (OS) compared to pancreatic body/tail cancer (6). In the selection of treatment strategies, radical pancreaticoduodenectomy remains the only potential curative treatment for patients with PHC (7, 8), but few patients are suitable for surgical treatment because of distant metastases or local invasion (9).

The accurate categorization of disease severity is pivotal for determination of appropriate and effective treatment (10). Recent studies have proposed various nodal staging systems for overall survival (OS) prediction (11), among which the most widely accepted for risk stratification is the N staging system. However, the N staging system neglects the influence of lymph node dissection. Currently, novel nodal staging systems was developed and gradually applied based on ratio of metastatic to retrieval lymph nodes (LNR), the log odds of lymph node ratio (LODDS) and the log odds of negative lymph nodes/T stage (LONT) (12, 13). The comparisons of predictive performance of these nodal staging systems for PHC has produced a spectrum of inconsistent findings, and previous studies often grapple with several limitations of methodologies and sample size that can affect the interpretation of their results. It lacks large sample and multicenter studies to explore the prognostic value of different nodal staging systems.

Machine learning procedures that enable computer systems to learn statistical patterns from data during a training phase. This model can then be applied to data to autonomously execute tasks such as clustering, optimization, and prediction, without relying on task-specific instructions (14). Recently, machine learning procedures have gained widespread application and are extensively used in the construction of prognostic models, especially in liver cancer (15), gastrointestinal cancer (16), and cervical cancer (17). In pancreatic cancer, machine learning procedures are also extensively used to identify pivotal biomarkers and develop prognostic model. Li et al. constructed a machine learning histamine-related signatures to reveal the prognosis of pancreatic cancer (18). Wu et al. developed an interpretable machine learning model, integrating radiomic features and clinicopathological factors, to predict the early recurrence in patients with post-surgery (19).

Herein, we obtained the resectable PHC cohort from the Surveillance, Epidemiology, and End Results (SEER) database to compare prognostic performance of the four nodal staging systems. Another large-sample multicenter resectable PHC cohort was retrospectively collected from three Chinese institutions to validate accuracy and generalizability. In addition, based on optimal nodal staging systems, a novel machine learning model for PHC was developed, interpreted and validated.

## 2 Materials and methods

### 2.1 Patients characteristics, demographics and study design

**Figure 1:**
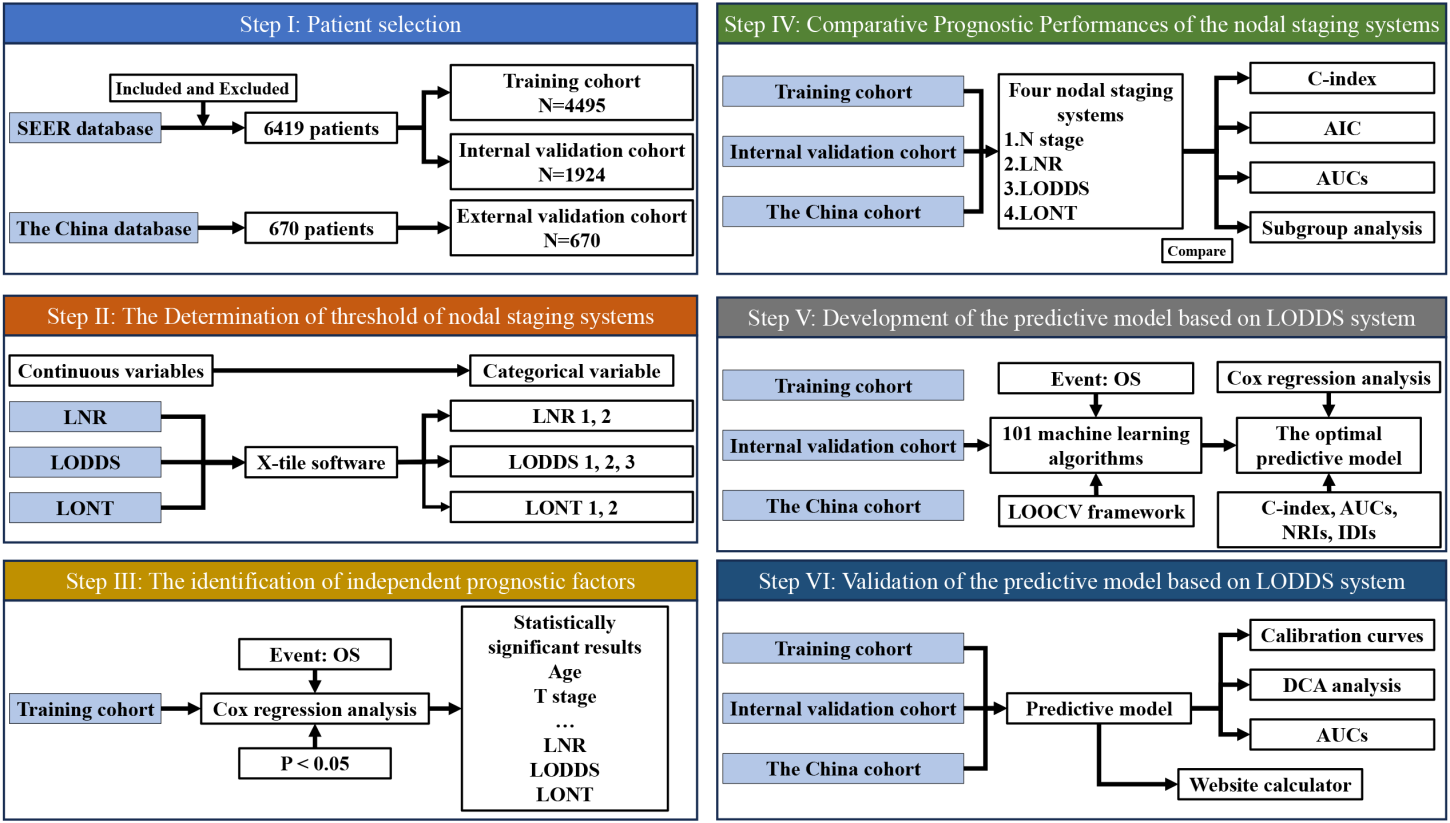
Flow diagram of the study population and design. LNR, The ratio of metastatic to retrieved nodes; LODDS, The log odds of lymph node ratio; LONT, The log odds of negative lymph nodes/T stage; OS, Overall survival; AIC, Akaike information criterion; ROC, Receiver operating characteristic; NRI, Net reclassification improvement; IDI, Integrated discrimination improvement; DCA, Decision curve analysis.

The resectable PHC patient characteristics were extracted from the SEER database of the National Cancer Institute (http://seer.cancer.gov/). The inclusion and exclusion criteria have been introduced in detail in our previous study (20). The AJCC staging was converted from the 7^th^ to the 8^th^ edition based on the tumor size, number of metastatic lymph nodes (MLNs) and distant metastasis for subsequent analysis. A large-sample, multicenter cohort including resectable PHC patients was collected from three Chinese institutions for external validation. The patients were follow-up by telephone or outpatient clinic interview. The variable selection is in accordance with the SEER cohort. This study was approved by the Ethics Committee of the PLA General Hospital. Written informed consent was obtained from all patients. The Flow diagram of the study population and design was shown in **Figure 1**.

Next, in this study, the LNR was defined as the ratio of the number of MLNs to the number of total retrieval lymph nodes (RLNs). The LODDS were determined as log [(MLNs + 0.5)/(negative lymph nodes + 0.5)]. The LONT was calculated as log odds of negative lymph nodes/T stage. According to the previous studies, the threshold of LNR was identified as 0.20, while the other optimal threshold of nodal staging systems was calculated using the X-tile software (version 3.6.1).

Then, the SEER cohort were randomly divided into the training and internal validation cohort at a ratio of 7:3 using “caret” package in R software (version 4.3.3). The training cohort was used to evaluate performance of different nodal staging systems predicting OS, and the performance was verified and generalized in the internal and external validation cohort.

Finally, based on optimal nodal staging systems, a novel machine learning model to predict OS for PHC was developed, interpreted and validated. Furthermore, a website calculator was established to facilitate the application of our predictive model more conveniently.

### 2.2 Statistical analyses

R (version 4.3.3) and SPSS (version 26.0) were used to complete all statistical analyses in the present study. X-tile software (version 3.6.1) is a common and practical approach for exploring optimal thresholds and thus is used to obtain the optimal cut-off values of partial nodal staging systems. Continuous variables are reported as median (interquartile range) and were compared by Student’s t test, and categorical variables are reported as counts and proportions and were analyzed by chi-squared test for comparisons among groups. Univariate Cox regression analysis and 10 machine learning-based integrative procedures, including random survival forest (RSF), CoxBoost, stepwise Cox, least absolute shrinkage and selection operator (Lasso), Ridge, elastic net (Enet), survival support vector machines (survival-SVMs), generalized boosted regression models (GBMs), supervised principal components (SuperPC) and partial least Cox (plsRcox), are implemented for identification of the prognostic factors. Through comparing these 101 machine learning combinations, the optimal model can be selected accordingly. Then, multivariate Cox regression analysis was carried out for construction of predictive model. Kaplan-Meier (KM) survival analysis and the log-rank test were performed to depict the capability of individual variables to discriminate overall survival (OS). The concordance index (C-index) was calculated to evaluate the discrimination of these nodal staging systems, Akaike Information Criterion (AIC) value, and time-dependent receiver operating characteristic (time-ROC) curves within 60 months were created to compare their predictive capability. The net reclassification improvement (NRI) and integrated discrimination improvement (IDI) were calculated to evaluate the improvement of the model prediction compared to the AJCC staging system. Finally, the performance and clinical benefits of predictive model was evaluated by calibration curve and decision curve analysis (DCA). The main utilized R packages were “rms”, “nricens”, “survival”, “Cschange”, “timeROC”, and so forth. A two-sided p < 0.05 was considered statistically significant.

## 3 Results

### 3.1 Patient demographics and characteristics

In the SEER cohort, a total of 6419 resectable PHC patients met the criteria after consulting the CS Schema v0204+, which included 4495 patients in the training cohort and 1924 patients in the internal validation cohort. In the external validation cohort, 670 resectable PHC patients was collected for validation and generalization. Among all resectable PHC patients, most of them were 60–80 years old (66.43%), male (55.15%), Race white (82.69%). The majority of them with AJCC II stage (43.26%), T2 stage (62.21%), and N1 stage (40.71%). Furthermore, the most common tumor differentiation was moderate (52.34%). With regard to the treatment, there were 4801 (74.79%) patients who received chemotherapy. There was no statistical difference in the demographics and characteristics between the training and internal validation cohort. In the external validation cohort, similarly, most of them were 60–80 years old (49.55%), male (55.67%). Moreover, the majority of them with AJCC I stage (52.69%), T2 stage (61.94%), and N0 stage (25.52%). In addition, the most common tumor differentiation was moderate (47.31%). With regard to the treatment, there were 283 (42.24%) patients who received chemotherapy. The baseline demographics and characteristics are shown in **Tables 1**, **2**.

**Table 1:** Baseline demographics and characteristics of patients in SEER cohort.

Variables	All patients	Training cohort	Internal validation cohort	*P* value
	n=6419	n=4495	n=1924	
Age, years, <40/40-60/60-80/≥80	44(0.69)/1464(22.80)/4264(66.43)/647(10.08)	38(0.85)/1010 (22.67)/2984(66.38)/463(10.30)	6(0.31)/454(23.60)/1280(66.53)/184(9.56)	0.070
Male, N (%)	3283(51.15)	2269(50.48)	1014(52.70)	0.102
Race, N (%) White/Black/Others	5308(82.69)/597(9.30)/514(8.01)	3712(82.58)/411(9.14)/372(8.28)	1596(82.95)/186(9.67)/142(7.38)	0.413
AJCC stage, N (%) I/II/III	1560(24.31)/2777(43.26)/2082(32.43)	1100(24.47)/1931(42.96)/1464(32.57)	460(23.91)/846(43.97)/61832.12)	0.749
T stage, N (%) T1/T2/T3/T4	1032(16.08)/3993(62.21)/1162(18.10)/232(3.61)	739(16.44)/2798(62.25)/786(17.49)/172(3.82)	293(15.23)/1195(62.11)/376(19.54)/60(3.12)	0.100
N stage, N (%) N0/N1/N2	1882(29.32)/2613(40.71)/1924(29.97)	1321(29.39)/1823(40.55)/1351(30.06)	561(29.16)/790(41.06)/573(29.78)	0.931
Differentiation, N (%), Low/moderate/poor/Undifferentiated	698(10.88)/3360(52.34)/2314(36.05)/47(0.73)	476(10.59)/2355(52.39)/1638(36.44)/26(0.58)	222(11.54)/1005(52.23)/676(35.14)/21(1.09)	0.086
Max tumor size, median (IQR), cm	3.0[2.5-4.0]	3.0[2.4-4.0]	3.0[2.5-4.0]	0.730
Chemotherapy, yes vs no	4801(74.79)	3362(74.79)	1439(74.79)	0.999
RLNs, median (IQR), count	17[12-24]	17[12-24]	17[12-23]	0.409
MLNs, median (IQR), count	2[0-4]	2[0-4]	2[0-4]	0.689
NLNs, median (IQR), count	14[9-21]	14[9-21]	14[9-20]	0.476
LNR, N (%), ≤0.20/>0.20	4420(68.86)/1999(31.14)	3086(68.65)/1409(31.35)	1334(69.33)/590(30.67)	0.590
LODDS, N (%), I/II/III	2411(37.56)/2826(44.03)/1182(18.41)	1686(37.51)/1980(44.05)/829(18.44)	725(37.68)/846(43.97)/353(18.35)	0.990
LONT, N (%), I/II	3318(51.69)/3101(48.31)	2310(51.39)/2185(48.61)	1008(52.39)/916(47.61)	0.758

Data are presented as n (%) or median (IQR).

RLNs, Retrieval lymph nodes; MLNs, Metastatic lymph nodes; NLNs, Negative lymph nodes; LNR, The ratio of metastatic to retrieved nodes; LODDS, The log odds of lymph node ratio; LONT, The log odds of negative lymph nodes/T stage.

**Table 2:** Baseline demographics and characteristics of patients in China cohort.

Variables	All patients
	n=670
Age, years, <40/40-60/60-80/≥80	37(5.52)/294(43.88)/332(49.55)/7(1.05)
Male, N (%)	373(55.67)
Race, N (%) White/Black/Others	–
AJCC stage, N (%) I/II/III	353(52.69)/250(37.31)/67(0.10)
T stage, N (%) T1/T2/T3/T4	128(19.10)/415(61.94)/101(15.07)/26(3.89)
N stage, N (%) N0/N1/N2	458(68.36)/171(25.52)/41(6.12)
**Differentiation, N (%), Low/moderate/poor**	112(16.72)/317(47.31)/241(35.97)
Max tumor size, median (IQR), cm	3.0[2.5-4.0]
**Chemotherapy, no vs yes**	283(42.24)
ELNs, median (IQR), count	11[10-15]
MLNs, median (IQR), count	0[0-1]
NLNs, median (IQR), count	11[9-14]
LNR, N (%), ≤0.20/>0.20	612(91.34)/58(8.66)
LODDS, N (%), I/II/III	464(69.25)/179(26.72)/27(4.03)
LONT, N (%), I/II	416(62.09)/25437.91)

RLNs, Retrieval lymph nodes; MLNs, Metastatic lymph nodes; NLNs, Negative lymph nodes; LNR, The ratio of metastatic to retrieved nodes; LODDS, The log odds of lymph node ratio; LONT, The log odds of negative lymph nodes/T stage; OS, Overall survival.

### 3.2 The determination of threshold of nodal staging systems

In the present study, the LNR ranged from 0 to 1, the LODDS ranged from -2.16 to 1.81, and the LONT ranged from 0 to 1.83 (**Supplementary Figure 1**). Based on the threshold, LNR was grouped into LNR1 (LNR ≤ 0.20) and LNR2 (LNR > 0.20), LODDS was classified into LODDS1 (-2.16 ≤ LODDS < -1.01), LODDS2 (-1.01 < LODDS ≤ -0.29), and LODDS3 (LODDS > -0.29), LONT was divided into LONT1 (LONT < 0.58) and LONT2 (LONT ≥ 0.58).

### 3.3 The identification of independent prognostic factors

In univariate Cox regression analysis, the interaction variables showed a statistically significant for age, AJCC staging, T stage, maximum tumor size, N stage, tumor differentiation, chemotherapy, MLNs, NLNs, LNR, LODDS, LONT (all p value < 0.05, **Table 3**). No statistically significant results were observed if stratified for sex and race (all p value > 0.05, **Table 3**). To avoid losing prognostic information and collinear contradiction, we performed four times multivariate Cox regression analysis. The results revealed four nodal staging systems could be deemed as independent prognostic factors (p value < 0.001, **Supplementary Table 1**). The KM curves depicted that all four nodal staging systems managed to significantly discriminate the OS of PHC patients (**Supplementary Figure 2**).

**Table 3:** Univariable Cox regression analysis for overall survival of patients with PHC in training cohort.

Variables	Univariable		*P* value
	B	HR (95%CI)	
Age, years			
<40	reference		
40-60	-0.079	0.924(0.624-1.368)	0.693
60-80	0.060	1.062(0.721-1.563)	0.762
≥80	0.417	1.518(1.018-2.263)	**0.040**
Sex			
Male vs Female	0.027	1.028(0.955-1.106)	0.466
Race			
Black vs White	-0.018	0.982(0.865-1.116)	0.783
Others vs White	0.053	1.054(0.921-1.207)	0.446
AJCC stage			
II vs I	0.469	1.598(1.446-1.767)	**<0.001**
III vs I	0.779	2.179(1.965-2.416)	**<0.001**
T stage			
T2 vs T1	0.423	1.526(1.368-1.704)	**<0.001**
T3 vs T1	0.698	2.011(1.765-2.291)	**<0.001**
T4 vs T1	0.655	1.926(1.571-2.361)	**<0.001**
Maximum tumor size, cm	0.008	1.009(1.007-1.010)	<0.001
N stage			
N1 vs N0	0.405	1.499(1.364-1.647)	**<0.001**
N2 vs N0	0.724	2.062(1.869-2.274)	**<0.001**
Tumor differentiation			
Moderate vs Low	0.337	1.400(1.219-1.608)	**<0.001**
Poor vs Low	0.717	2.049(1.780-2.359)	**<0.001**
Undifferentiated vs Low	0.946	2.576(1.631-4.068)	**<0.001**
Radiotherapy			
** Yes vs no**	-0.343	0.709(0.655-0.768)	**<0.001**
Chemotherapy			
** Yes vs no**	-0.653	0.520(0.480-0.564)	**<0.001**
MLNs, median (IQR), count	0.066	1.068(1.059-1.078)	<0.001
NLNs, median (IQR), count	-0.016	0.984(0.980-0.988)	<0.001
LNR			
≤0.20/>0.20	0.587	1.799(1.665-1.943)	**<0.001**
LODDS			
II vs I	0.462	1.588(1.458-1.729)	**<0.001**
III vs I	0.818	2.267(2.045-2.513)	**<0.001**
LONT			
II vs I	-0.396	0.673(0.625-0.725)	**<0.001**

P values < 0.05 indicate a significant difference between the two groups are given in bold.

MLNs, Metastatic lymph nodes; NLNs, Negative lymph nodes; LNR, The ratio of metastatic to retrieved nodes; LODDS, The log odds of lymph node ratio; LONT, The log odds of negative lymph nodes/T stage; PHC, Pancreatic head cancer.

### 3.4 Comparative prognostic performances of the four nodal staging systems for OS prediction

**Figure 2:**
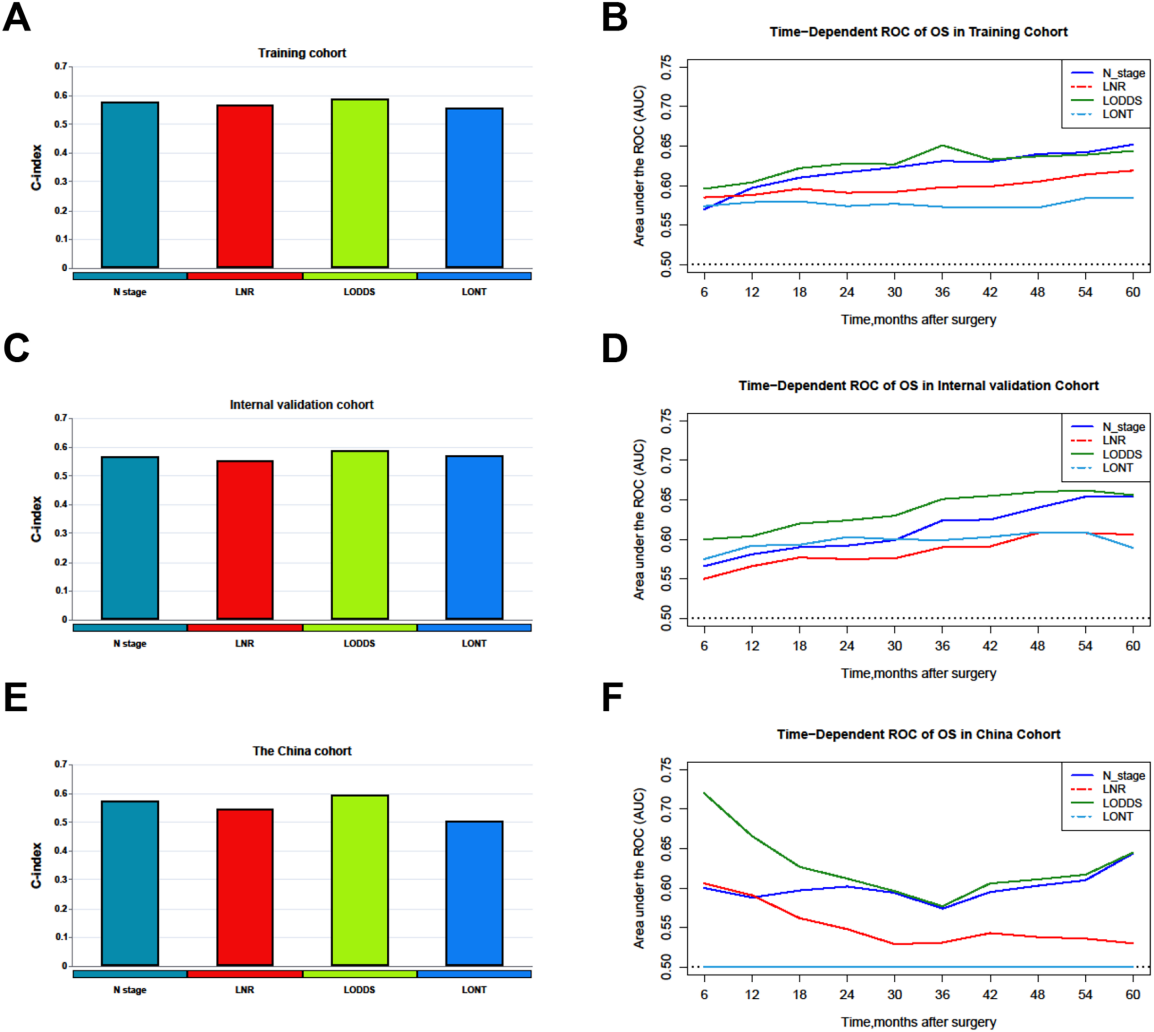
Comparison of the C-index and time-dependent AUCs among nodal staging systems. **(A, B)** Training cohorts. **(C, D)** Internal validation cohort. **(E, F)** The China cohort. LNR, The ratio of metastatic to retrieved nodes; LODDS, The log odds of lymph node ratio; LONT, The log odds of negative lymph nodes/T stage; OS, Overall survival; AUC, Area under the curve.

Comparisons of discriminability among four nodal staging systems were performed in the all cohorts. In the training cohort, LODDS has the highest C-index among all nodal staging systems, which is 0.589 (95% CI 0.578–0.600) and has best predictive capability than the N stage (C-index: 0.578, 95% CI 0.567–0.590, p = 0.005), LNR (C-index: 0.569, 95% CI 0.559–0.579, p < 0.001), and LONT (C-index: 0.559, 95% CI 0.549–0.569, p < 0.001). In the internal validation cohort, LODDS also has the highest C-index among all nodal staging systems, which is 0.590 (95% CI 0.573–0.607) and has best predictive capability than the N stage (C-index: 0.567, 95% CI 0.549–0.584, p < 0.001), LNR (C-index: 0.555, 95% CI 0.540–0.570, p < 0.001), and LONT (C-index: 0.573, 95% CI 0.558–0.589, p = 0.06). Similarly, in the external validation cohort, the LODDS has the highest C-index, which is 0.596 (95% CI 0.573–0.620), compare to the N stage (C-index: 0.575, 95% CI 0.551–0.599, p = 0.011), LNR (C-index: 0.546, 95% CI 0.529–0.563, p < 0.001), and LONT (C-index: 0.504, 95% CI 0.480–0.527, p < 0.001). The detailed information is shown in **Table 4**, **Figures 2A–C**. Next, we calculated the AIC values to verified the optimal predictive performance. The results showed that the LODDS has the lowest AIC value than others in all cohorts (**Table 4**). Then, the time-dependent area under the curves (AUCs) of the nodal staging systems for predicting OS within 60 months depict that LODDS has the highest AUC compared to others (**Figures 2D–F**). All the results indicated that LODDS has an excellent performance for lymph nodes stratification of resectable PHC patients.

**Table 4:** The discriminatory performance of nodal staging systems in predicting overall survival of patients with PHC.

Variables	Training cohort		Internal validation cohort		External cohort	
	C-index (95% CI)	AIC	C-index (95% CI)	AIC	C-index (95% CI)	AIC
N stage	0.578(0.567–0.590)	42739	0.567(0.549–0.584)	15755	0.575(0.551–0.599)	5423.9
LNR	0.569 (0.559–0.579)	42742	0.555(0.540–0.570)	15764	0.546(0.529–0.563)	5445.0
LODDS	0.589(0.578–0.600)	42699	0.590(0.573–0.607)	15706	0.596(0.573–0.620)	5420.4
LONT	0.559(0.549–0.569)	42847	0.573(0.558–0.589)	15755	0.504(0.480–0.527)	5472.7

LNR, The ratio of metastatic to retrieved nodes; LODDS, The log odds of lymph node ratio; LONT, The log odds of negative lymph nodes/T stage; AIC, Akaike information criterion; PHC, Pancreatic head cancer.

### 3.5 Comparative prognostic performances of the four nodal staging systems based on the number of RLNs

According to previous study and AJCC staging system, the recommended number of RLNs for pathologic examination is 12 (21, 22). The results of C-index and AUC values indicated that LODDS system has better predictive capability than others both in patients with < 12 and ≥ 12 RLNs for OS prediction (**Supplementary Table 2**), revealing that it was not affected by the number of RLNs.

### 3.6 Risk stratification ability of LODDS among clinicopathological characteristics

We subsequently revealed the risk stratification ability using the LODDS system among clinicopathological characteristics, including age, gender, tumor differentiation, AJCC staging, T stage, and chemotherapy, and found that the LODDS system has a good ability to distinguish the prognosis of PHC patients in gender, tumor differentiation, AJCC staging, and T stage subgroups (**Supplementary Figure 3**).

### 3.7 Development, interpretation and validation of the predictive model for OS based on LODDS system

Based on the results of univariate Cox analysis, the variables (Age, T stage, chemotherapy, tumor differentiation, and LODDS) were subjected to our machine learning-based integrative procedure to develop a consensus model. We fitted 101 machine learning combinations via the LOOCV framework and further calculated the C-index of each combination across the training, internal validation and external validation cohorts.

**Figure 3:**
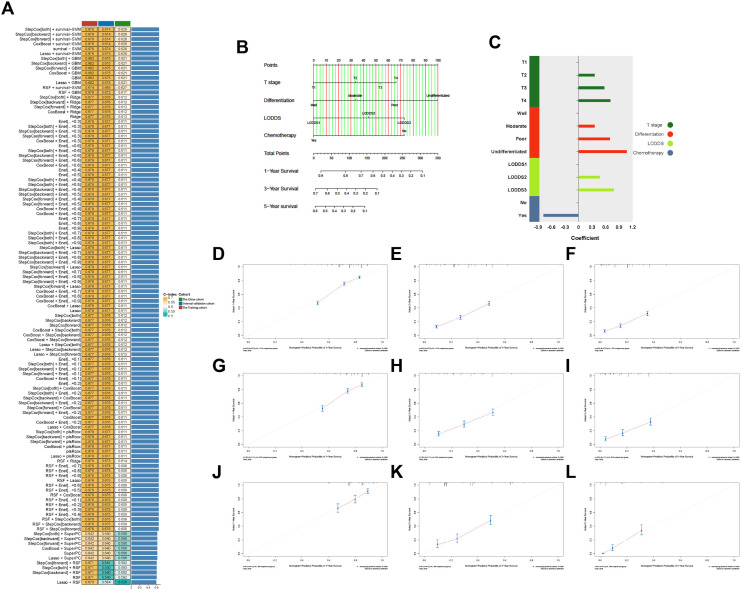
**(A)** Of 101 kinds of prediction models via LOOCV framework and further calculated the C-index of each model across all validation datasets. **(B)** The nomogram predicting OS of patients with PHC. **(C)** The coefficient of each variable included in predictive model. **(D–L)** Calibration curves showing the probability of 12-, 36-, and 60-month OS of the model prediction and the observed survival. **(D–F)** Training cohorts. **(G–I)** Internal validation cohort. **(J–L)** The China cohort. LODDS, The log odds of lymph node ratio; OS, Overall survival; PHC, Pancreatic head cancer.

Notably, the optimal model was a combination of stepwise Cox (direction = both) and survival−SVM with the highest average C-index (0.659), and this combination model had a related leading C-index both in internal validation and the external validation cohorts (**Figure 3A**). Then, a predictive model for OS was developed and validated via multivariable Cox analysis (**Figures 3B, C**). Next, the calibration curves of all cohorts for survival probability depicted that the model prediction had good consistency with the actual observation (**Figures 3D–L**).

**Figure 4:**
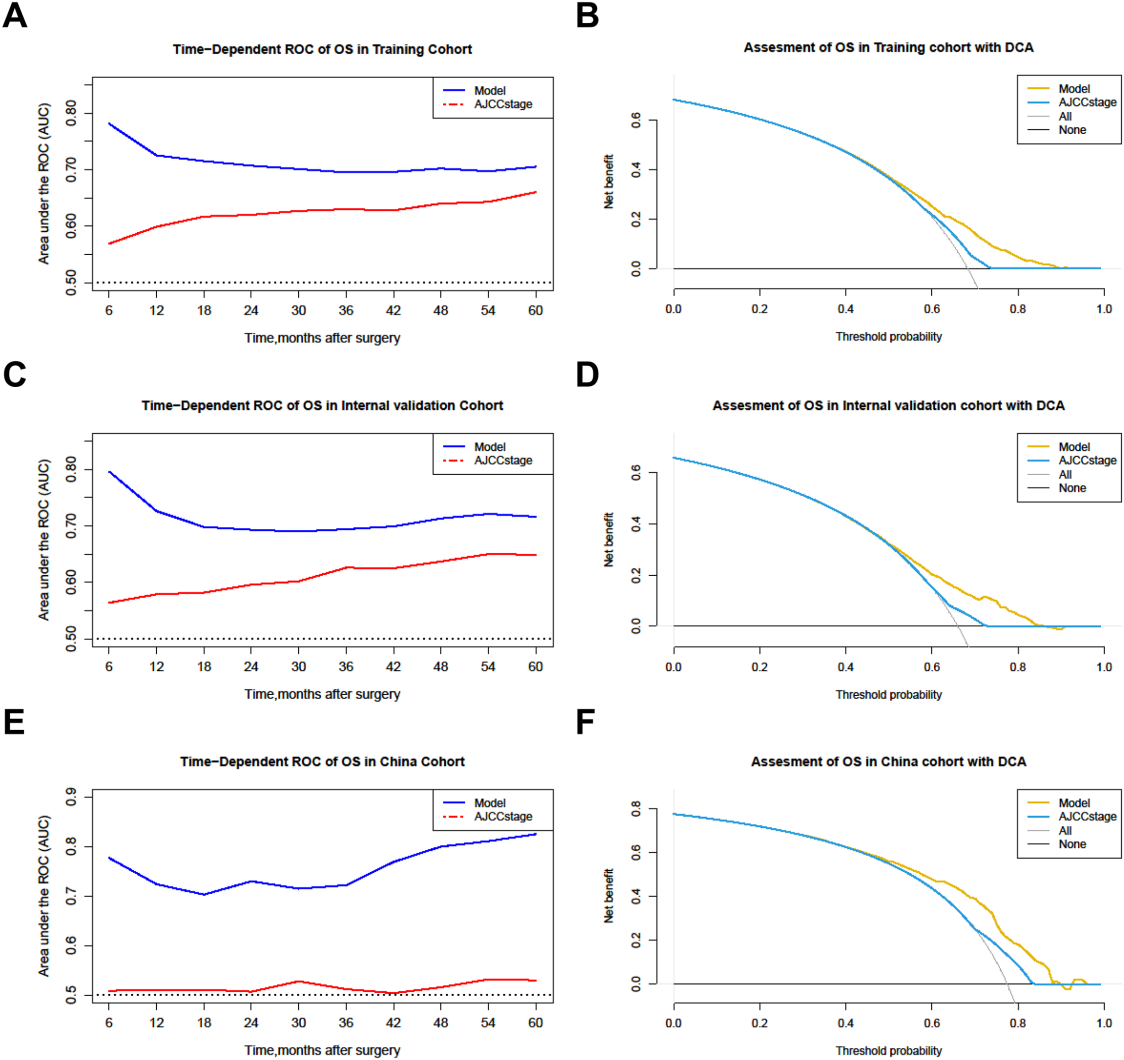
Comparison of the time-dependent AUCs and DCA between the model and the AJCC staging system. **(A, B)** Training cohorts. **(C, D)** Internal validation cohort. **(E, F)** The China cohort. OS, Overall survival; ROC, Receiver operating characteristic; DCA, Decision curve analysis.

For interpretation of the predictive model, the C-index, NRI, and IDI of the predictive model had better predictive capability than the AJCC staging system in all cohorts (**Table 5**), and the AUCs were significantly higher in all cohorts compared to those of the AJCC staging system (**Figures 4A–C**). All the results indicate that the predictive model had favorable discrimination for OS in PHC. Finally, DCA analysis demonstrated that the clinical utility of the present model was better, compared with the AJCC staging system in all cohorts (**Figures 4D–F**).

**Table 5:** The NRIs, IDIs, and C-index of the predictive model and AJCC 8^th^ staging system in overall survival prediction.

Index	Training cohort			Validation cohort			External cohort		
	Estimated	95%CI	P Value	Estimated	95%CI	P Value	Estimated	95%CI	P Value
NRI (vs AJCC 8^th^ staging system)									
For 12-month OS	0.262	0.225-0.360		0.287	0.209-0.362		0.223	0.138-0.352	
For 36-month OS	0.054	0.009-0.107		0.106	0.046-0.255		0.277	0.202-0.412	
For 60-month OS	0.108	0.069-0.197		0.141	0.085-0.280		0.398	0.153-0.559	
IDI (vs AJCC 8^th^ staging system)									
For 12-month OS	0.056		**<0.001**	0.061		**<0.001**	0.071		**<0.001**
For 36-month OS	0.064		**<0.001**	0.076		**<0.001**	0.152		**<0.001**
For 60-month OS	0.052		**<0.001**	0.062		**<0.001**	0.129		**<0.001**
C-index									
Predict model	0.675	0.664-0.686		0.675	0.658-0.693		0.664	0.638-0.691	
AJCC 8^th^ staging system	0.581	0.570-0.592		0.566	0.549-0.584		0.518	0.491-0.544	
Change	0.094	0.082-0.105	**<0.001**	0.109	0.088-0.015	**<0.001**	0.147	0.109-0.176	**<0.001**

P values < 0.05 indicate a significant difference between the two groups are given in bold.

NRI, Net reclassification improvement; IDI, Integrated discrimination improvement; OS, overall survival; CI, Confidence Interval.

### 3.8 Online access of the predictive model

**Figure 5:**
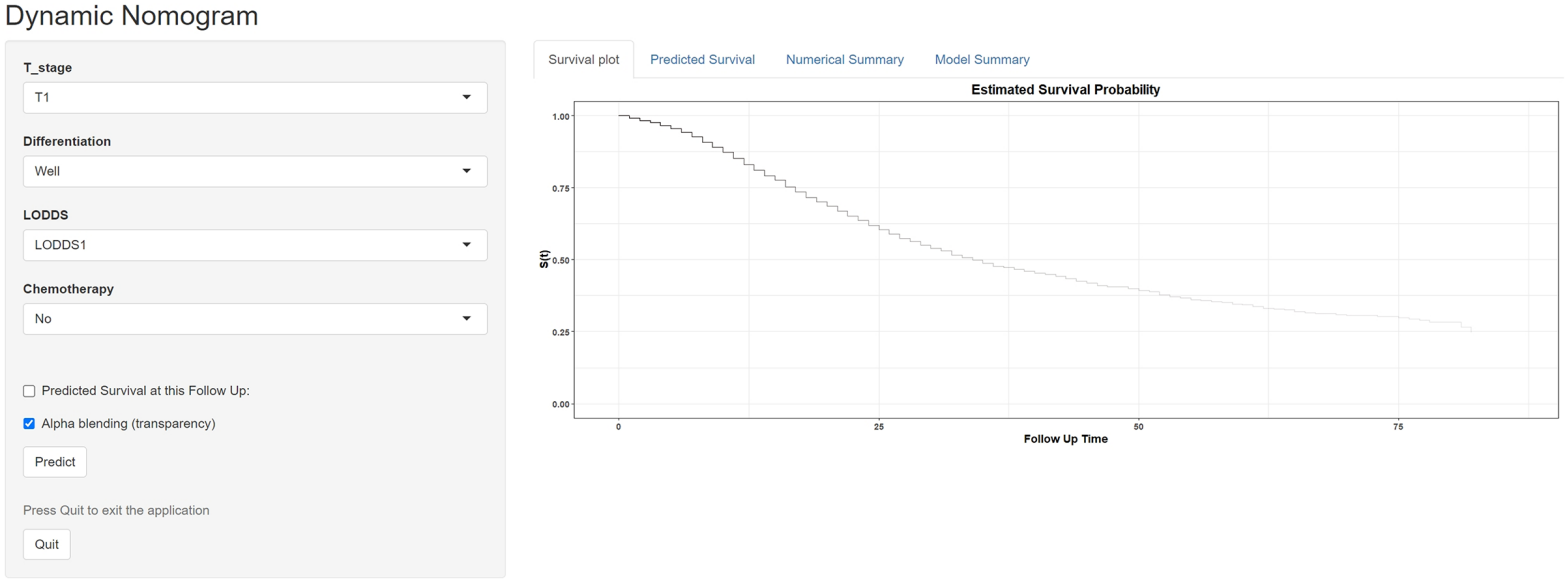
Application of website calculator for online access of the predictive model.

To facilitate the application of our machine learning-based nomogram more conveniently, we developed and established a website calculator (https://vs-prediction.shinyapps.io/OS-PHC-prediction-tool/, **Figure 5**). Clinical physicians can calculate the corresponding survival probabilities by entering the demographics and characteristics of patients with PHC.

## 4 Discussion

The accurate evaluation of lymph nodes metastases is pivotal for determination of appropriate and effective treatment. Previous studies have proposed various nodal staging systems, but their predictive value in forecasting the overall survival (OS) of patients with PHC remains to be determined (23). This study investigated the current nodal staging systems N stage, LNR, LODDS and LONT, explored their optimal threshold, and compared their prognostic performance using the large-sample, multicenter data from the SEER and the China databases. Differences in prognostic performance of the nodal staging systems were also investigated in each subgroup according to clinicopathological characteristics. Our results proved that the LODDS is the relatively best lymph node metastasis classification system compared to others. To our knowledge, this is the first study comparing the current four nodal staging systems in PHC patients, and further developing a novel predictive model based on LODDS and clinicopathological factors to predict the OS of resectable PHC patients.

The oncologic outcomes of PHC are very important in guiding the selection of treatment methods, and among these, lymph node metastasis is significantly prognostic factor for predicting survival of patients (24). Thus, effectively distinguishing the extent of lymph nodal metastasis is crucial. The most widely accepted for risk stratification is the N staging system. The N staging system stratified patients according to the number of MLN. In the light of AJCC staging system, the patients without MLN were referred as N0 stage, with 1–3 MLNs were referred as N1 stage, and ≥4 MLNs were referred as N2 stage (24). It has been applied to evaluate the extent of nodal metastasis of various malignant tumors. However, assessing prognosis solely based on the N staging system or MLN may be biased due to incomplete lymph node dissection or inadequate histopathological examination (25, 26). In recent years, a variety of lymph node staging systems have gradually emerged. LNR, an indicator reflecting the status of lymph node burden, holds significant importance in predicting prognosis of several tumors and less susceptibility to the influence of the number of RLNs (27, 28). Previous studies suggest that LNR is a promising biomarker, similarly, our results also verified that LNR may provide good prognostic stratification (12). The LODDS system has the advantage of good discrimination in patients with fewer number of RLNs and those without lymph node metastasis (29). The LODDS system is proposed to overcome the disadvantage of LNR and used for prognosis predicting and clinical stratification in tumour (30). It offers a more nuanced assessment of lymph node involvement than traditional staging methods. In addition, a new predictive indicator for assessing the lymph node status, known as LONT, has been devised for patient stratification. LONT can reflect both the stage of disease and the extent of lymph node dissection. In previous studies, LONT has been used for numerous tumors prognosis prediction with good accuracy (31, 32). Our study showed similar findings in prognostic performance of these nodal staging systems. They have all been proven to be independent prognostic factors for OS prediction of tumors including PHC.

Here, we comprehensively calculated and analyzed the LNR, LODDS, and LONT values of each participants using multicenter registry data from SEER and the China databases, and then obtained different subgroups based their cut-off values. The results of our analyses, including the C-index, AICs, AUCs within 60 months, showed that LODDS had best accuracy for predicting survival than other nodal staging systems in all cohorts. Riediger et al. study found LNR and LODDS are superior to the classical nodal status in predicting prognosis in resectable PHC, while LODDS has not shown advantage over LNR in their series of 409 patients (33). In our study, we demonstrated the LODDS could be considered as optimal indicator for nodal risk stratification and OS prediction of PHC patients. This result can provide additional evidence to prove the predictive performance of LODDS in patients with resectable PHC based on a larger-sample, multicenter cohort.

The number of RLNs for pathologic evaluation plays a pivotal role in assessment of lymph node status. According to previous studies, the recommended number of RLNs for pathologic evaluation is 12 (22). However, the detection capability for lymph nodes varies due to the different surgical approaches and strengths of medical institutions, there is an urgent need for a node staging system that can evaluate patients without considering the number of RLNs. Previous studies have proposed the LNR or LODDS may be superior to N staging system in patients with fewer RLNs (29, 34). This study showed similar findings. The LODDS system yielded an equivalent C-index and AUCs in patients with < 12 and ≥ 12 RLNs, and higher C-index and AUCs than other nodal staging systems in patients with < 12 and ≥ 12 RLNs. Therefore, LODDS could be considered as a more adequate nodal staging system for PHC because it is almost unaffected by the number of RLNs. The LODDS can be promising indicator of assessment of lymph nodal status, and the predictive model based on LODDS can be reproduced using a simple data collection, making it attractive for clinical translation and implementation in medical institutions at different levels.

To construct effective model based on LODDS for clinical application, the machine learning-based integrative procedure was implemented in the study. In the current times, machine learning procedures have gained widespread application and are extensively used in the construction of prognostic models, especially in various cancers (18, 19). In pancreatic cancer. Ren et al. constructed a novel 4 pancreatic cancer-related protein signatures model based on machine learning (35). Lee et al. used the machine learning procedures to identify and develop the microbiome markers-based model for diagnosis of pancreatic cancer (36). Additionally, Zhu et al. developed an interpretable machine learning model for predicting early liver metastasis after pancreatic cancer surgery (37). In the present study, we fitted 101 kinds of prediction models via the LOOCV framework in the training cohort to optimize variable selection with the highest average C-index. And further validations in the internal and external validation cohorts. The advantage of the comprehensive procedures is that it is based on various machine learning algorithms and their combinations to fit a model with consistent performance on the prognosis of PHC. The strategy can further reduce the dimensionality of variables, making the model more simplified and translational (38). Furthermore, AUCs, C-index, NRIs, and IDIs analysis suggested that the predictive model maintained the favorable discrimination and stable performance for OS prediction in all cohorts, which indicated great potential for the clinical application of predictive model. Previous study also developed many predictive model or risk score for OS prediction of PHC patients. Among these models, a limited number have been successfully integrated into clinical practice, and an even smaller subset has undergone rigorous external validation (20, 39, 40). Shi et al. established a nomogram to predict the prognosis of pancreatic cancer patients underlying surgery but did not conduct external validation (41). In addition, Peng et al. developed a nomogram to assess the survival period of postoperative pancreatic cancer patients and implemented a single center validation (42). In our study, the prognostic and machine learning analysis demonstrated that our predictive model was a commendable indicator of OS in PHC and had a better extrapolation possibility using multicenter registry data. The calibration curves for survival probability depicted that the model prediction had good consistency with the actual observation, and DCA analysis demonstrated that the good clinical utility of the present model. Finally, a convenient website calculator (https://vs-prediction.shinyapps.io/OS-PHC-prediction-tool/) to facilitate the application of our machine learning nomogram. Clinical physicians can calculate the corresponding survival probabilities by entering the clinical data embedded within Electronic Health Record (EHR) systems or accessed via hospital intranet portals. This facilitates its use in risk stratification, decision-making, and patient counseling, thereby seamlessly integrating data-driven prognostication into routine clinical practice.

The innovation of this study can be highlighted in two main aspects. Firstly, in the present study, the nodal staging system LODDS is the optimal prognostic factor with good performance comparable to the others. The LODDS was applicable regardless of the numbers of RLN. Second, the machine-learning model could effectively predict OS for resectable PHC patients.

Several limitations should be acknowledged in the present study. Firstly, all of the samples from this study were retrospective, and future availability of LODDS and validation of predictive model should be performed in prospective cohort. Secondly, the cut-offs were derived from the SEER cohort, their prognostic value was limited in the independent external cohort. However, the consistent performance across distinct populations significantly mitigates the concern of overfitting and supports the generalizability of these thresholds. Thirdly, difficulty in immediate calculation of LODDS restricted clinical application of the LODDS system in real-world practice. A simple calculator is worth developing in future study. Next, different demographic characteristics in SEER and Chinese cohorts may result in limited outcomes. However, the LODDS and predictive model maintained predictive accuracy across two geographically and clinically distinct populations, suggesting good generalizability. Finally, we lacked some routinely clinicopathological characteristics such as serological indicators, R0 resection rate and so forth. The absence of this information may have affected the factors needed in our model. Further verification and more clinicopathological characteristics collection are needed to optimize its application.

## 5 Conclusion

In conclusion, nodal staging system LODDS is an optimal prognostic indicator that can reflect the lymph nodal status with good performance. It could effectively predict OS for resectable PHC patients without considering the numbers of RLN. The machine learning model showed good predictive ability and could assist clinicians in formulating individualized treatment strategies.

## Data Availability

The raw data supporting the conclusions of this article will be made available by the authors, without undue reservation.
