# Supplementary material for: Predictive value of current nodal staging systems and development of machine learning nomogram for resectable pancreatic head cancer: a population-based study and multicenter validation
Source: Front Immunol. 2025 Dec 2;16:1639186. doi: 10.3389/fimmu.2025.1639186 (PMC12705621; doi:10.3389/fimmu.2025.1639186)
Supplement: Supplementary file 1 [file Table1.docx]

**Supplementary material**

**Title：Predictive value of current nodal staging systems and development of machine learning nomogram for resectable pancreatic head cancer: a population-based study and multicenter validation**

**Supplementary Figure:**


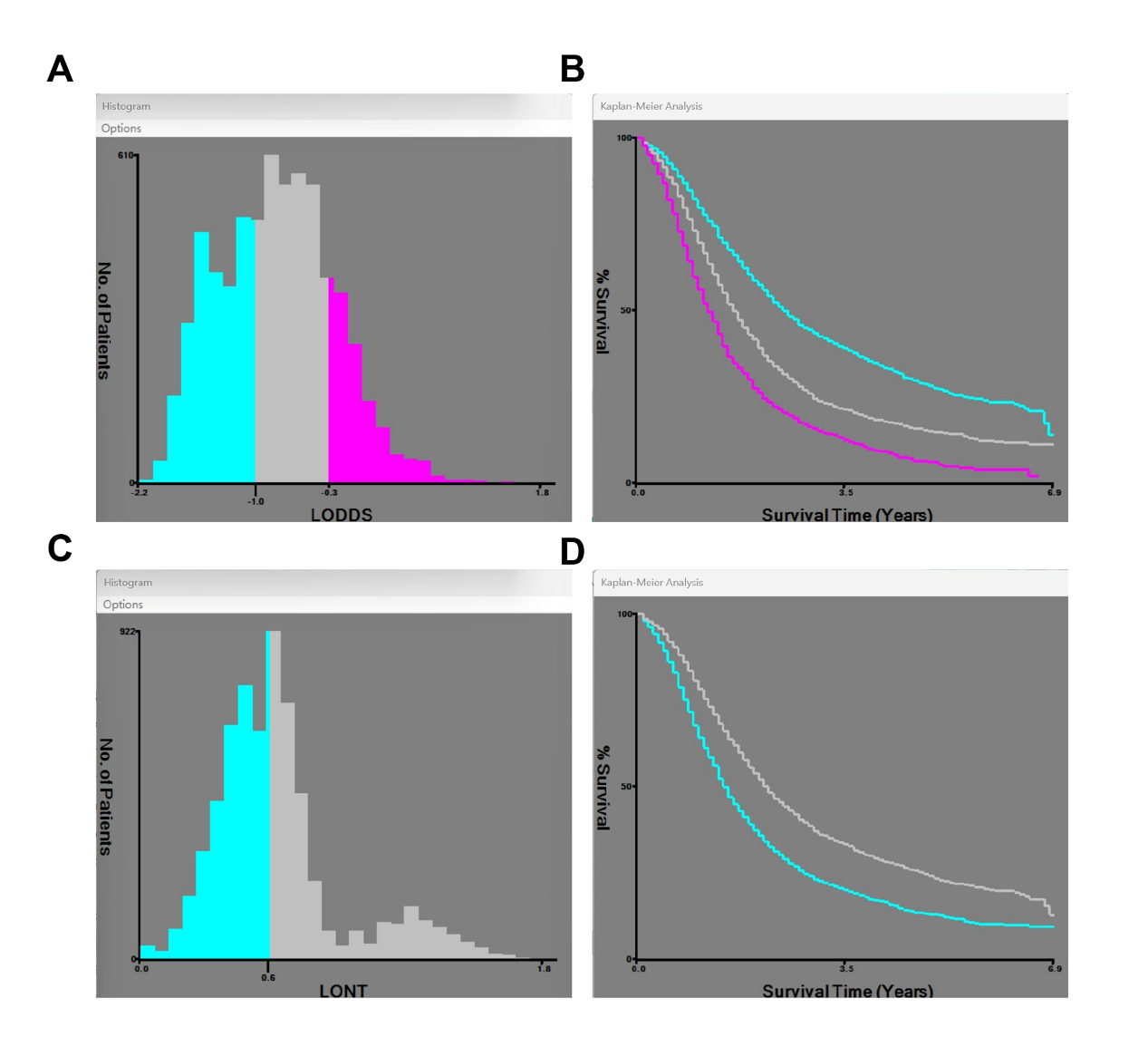


Supplementary Figure 1: The optimal cut-off value of LODDS and LONT via X-tile software. (A-B) LODDS. (C-D) LONT. Abbreviations: LODDS: The log odds of lymph node ratio; LONT: The log odds of negative lymph nodes/T stage.


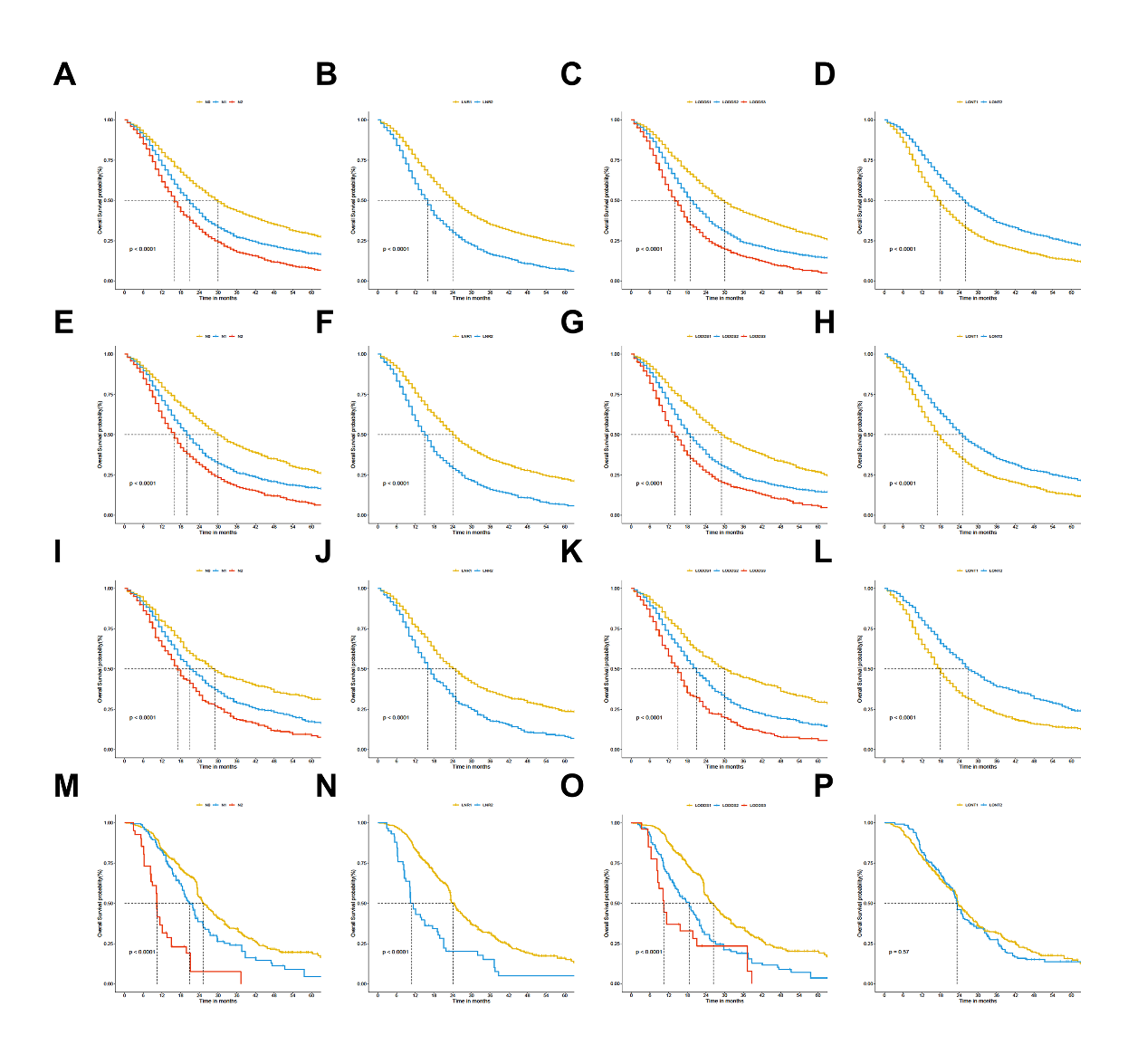


Supplementary Figure 2: The Kaplan-Meier curves of overall survival for patients and by stratified by four nodal staging system across all cohorts. (A-D) The total SEER cohorts. (E-H) Training cohorts. (I-L) Internal validation cohort. (M-P) The China cohort. Abbreviations: LNR: The ratio of metastatic to retrieved nodes; LODDS: The log odds of lymph node ratio; LONT: The log odds of negative lymph nodes/T stage.


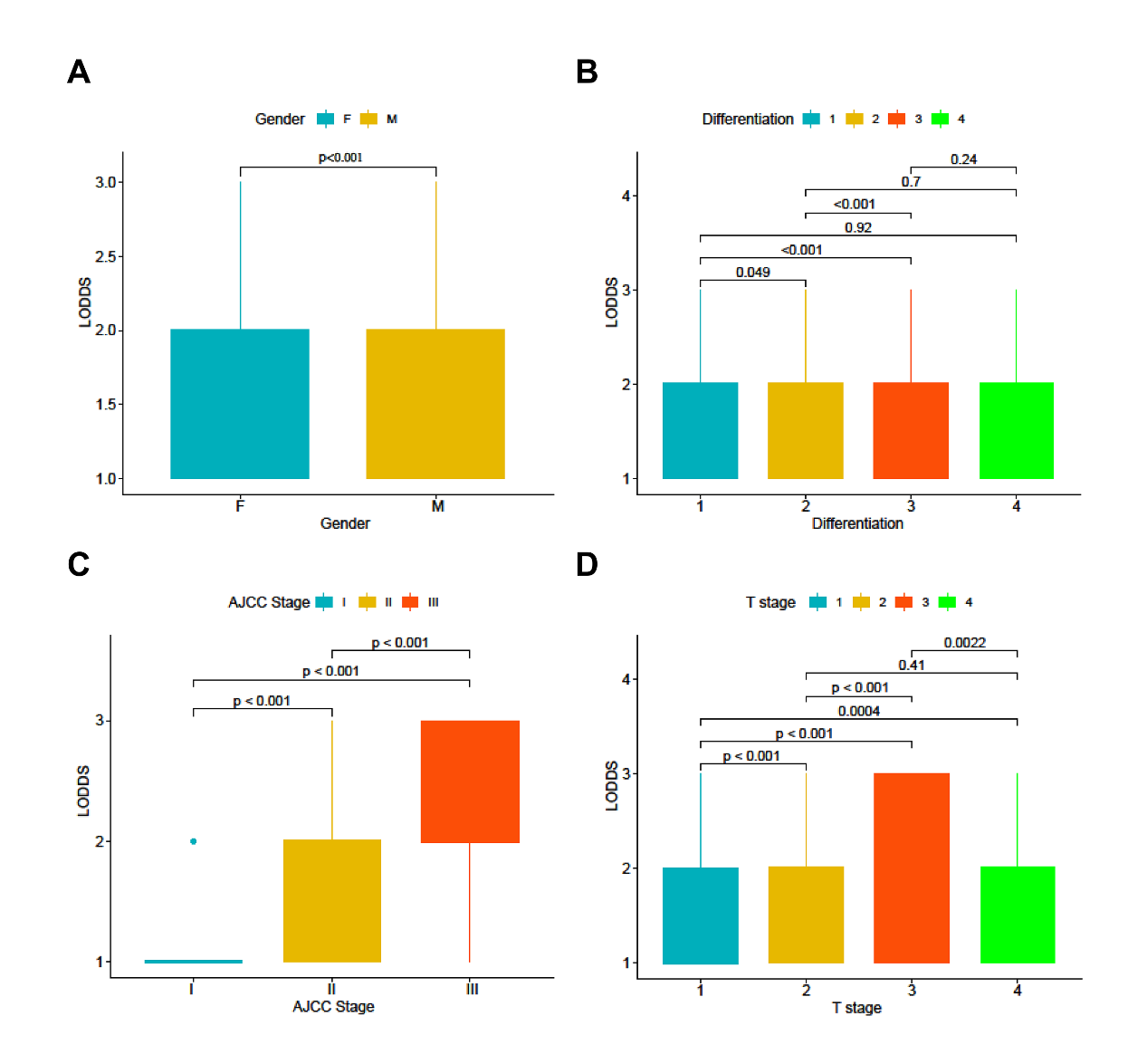


Supplementary Figure 3: Correlation of LODDS with clinical characteristics. (A) Gender. (B) Differentiation. (C) AJCC stage. (D) T stage. Abbreviations: LODDS: The log odds of lymph node ratio.

**Supplementary Table:**

| **Supplementary Table 1. Multivariable Cox regression analysis for overall survival of patients with PHC in training cohort** | | | |
| --- | --- | --- | --- |
| **Variables** | **Univariable** | | ***P* value** |
|  | **B** | **HR (95%CI)** |  |
| Age, years |  |  |  |
| 40-60 | 0.059 | 1.060(0.715-1.573) | 0.771 |
| 60-80 | 0.114 | 1.121(0.760-1.653) | 0.565 |
| ≥80 | 0.307 | 1.360(0.911-2.030) | 0.133 |
| T stage |  |  |  |
| T2 vs T1 | 0.340 | 1.406(1.257-1.572) | **<0.001** |
| T3 vs T1 | 0.559 | 1.749(1.531-1.998) | **<0.001** |
| T4 vs T1 | 0.655 | 1.926(1.569-2.365) | **<0.001** |
| Tumor differentiation |  |  |  |
| Moderate vs Low | 0.322 | 1.380(1.201-1.586) | **<0.001** |
| Poor vs Low | 0.659 | 1.933(1.677-2.228) | **<0.001** |
| Undifferentiated vs Low | 0.984 | 2.675(1.692-4.230) | **<0.001** |
| Chemotherapy, yes vs no | -0.745 | 0.475(0.436-0.516) | **<0.001** |
| N stage |  |  |  |
| N1 vs N0 | 0.418 | 1.518(1.380-1.671) | **<0.001** |
| N2 vs N0 | 0.691 | 1.995(1.804-2.207) | **<0.001** |
|  | | | |
| **Variables** | **Univariable** | | ***P* value** |
|  | **B** | **HR (95%CI)** |  |
| Age, years |  |  |  |
| 40-60 | -0.044 | 0.957(0.646-1.419) | 0.827 |
| 60-80 | 0.021 | 1.021(0.693-1.505) | 0.916 |
| ≥80 | 0.214 | 1.239(0.830-1.848) | 0.295 |
| T stage |  |  |  |
| T2 vs T1 | 0.376 | 1.456(1.302-1.628) | **<0.001** |
| T3 vs T1 | 0.612 | 1.844(1.616-2.106) | **<0.001** |
| T4 vs T1 | 0.680 | 1.974(1.608-2.423) | **<0.001** |
| Tumor differentiation |  |  |  |
| Moderate vs Low | 0.355 | 1.427(1.241-1.639) | **<0.001** |
| Poor vs Low | 0.697 | 2.008(1.743-2.314) | **<0.001** |
| Undifferentiated vs Low | 1.036 | 2.848(1.782-4.456) | **<0.001** |
| Chemotherapy, yes vs no | -0.715 | 0.489(0.450-0.532) | **<0.001** |
| LNR, ≤0.20/>0.20 | 0.555 | 1.742(1.611-1.884) | **<0.001** |
|  | | | |
| **Variables** | **Univariable** | | ***P* value** |
|  | **B** | **HR (95%CI)** |  |
| Age, years |  |  |  |
| 40-60 | 0.018 | 1.019(0.687-1.511) | 0.927 |
| 60-80 | 0.067 | 1.070(0.725-1.578) | 0.734 |
| ≥80 | 0.236 | 1.267(0.849-1.891) | 0.247 |
| T stage |  |  |  |
| T2 vs T1 | 0.348 | 1.416(1.267-1.584) | **<0.001** |
| T3 vs T1 | 0.560 | 1.751(1.533-2.000) | **<0.001** |
| T4 vs T1 | 0.692 | 1.997(1.627-2.451) | **<0.001** |
| Tumor differentiation |  |  |  |
| Moderate vs Low | 0.354 | 1.425(1.240-1.638) | **<0.001** |
| Poor vs Low | 0.682 | 1.977(1.716-2.279) | **<0.001** |
| Undifferentiated vs Low | 1.051 | 2.859(1.808-4.522) | **<0.001** |
| Chemotherapy, yes vs no |  |  | **<0.001** |
| LODDS |  |  |  |
| II vs I | 0.463 | 1.588(1.456-1.732) | **<0.001** |
| III vs I | 0.761 | 2.140(1.927-2.377) | **<0.001** |
|  | | | |
| **Variables** | **Univariable** | | ***P* value** |
|  | **B** | **HR (95%CI)** |  |
| Age, years |  |  |  |
| 40-60 | -0.096 | 0.908(0.613-1.346) | 0.633 |
| 60-80 | -0.056 | 0.946(0.642-1.394) | 0.777 |
| ≥80 | 0.153 | 1.165(0.780-1.738) | 0.455 |
| T stage |  |  |  |
| T2 vs T1 | 0.337 | 1.401(1.244-1.577) | **<0.001** |
| T3 vs T1 | 0.489 | 1.630(1.394-1.907) | **<0.001** |
| T4 vs T1 | 0.554 | 1.740(1.394-2.172) | **<0.001** |
| Tumor differentiation |  |  |  |
| Moderate vs Low | 0.380 | 1.462(1.272-1.680) | **<0.001** |
| Poor vs Low | 0.740 | 2.095(1.818-2.414) | **<0.001** |
| Undifferentiated vs Low | 1.069 | 2.914(1.842-4.610) | **<0.001** |
| Chemotherapy, yes vs no |  |  | **<0.001** |
| LONT, II vs I | -0.241 | 0.785(0.717-0.860) | **<0.001** |

P values < 0.05 indicate a significant difference between the two groups are given in bold.

Abbreviations: LNR: The ratio of metastatic to retrieved nodes; LODDS: The log odds of lymph node ratio; LONT: The log odds of negative lymph nodes/T stage; PHC: Pancreatic head cancer; HR: Hazard ratio.

| **Supplementary Table 2. Prognostic performance based on the number of RLNs in each nodal staging system** | | | | | | | | | | | | | |
| --- | --- | --- | --- | --- | --- | --- | --- | --- | --- | --- | --- | --- | --- |
|  |  | **Training cohort** | | | | **Internal validation cohort** | | | | **External cohort** | | | |
| **C-index** |  |  |  |  |  |  |  |  |  |  |  |  |  |
|  | | N stage | LNR | LODDS | LONT | N stage | LNR | LODDS | LONT | N stage | LNR | LODDS | LONT |
| **All patients** | | 0.578 | 0.569 | 0.589 | 0.559 | 0.567 | 0.555 | 0.590 | 0.573 | 0.575 | 0.546 | 0.596 | 0.504 |
| **RLN** | **< 12** | 0.576 | 0.569 | 0.580 | 0.534 | 0.572 | 0.538 | 0.572 | 0.559 | 0.532 | 0.520 | 0.561 | 0.503 |
|  | **≥12** | 0.585 | 0.567 | 0.590 | 0.561 | 0.578 | 0.557 | 0.590 | 0.557 | 0.614 | 0.570 | 0.629 | 0.516 |
| **AUCs at 3 years** |  |  |  |  |  |  |  |  |  |  |  |  |  |
| **All patients** |  | 0.631 | 0.598 | 0.651 | 0.573 | 0.624 | 0.590 | 0.651 | 0.599 | 0.574 | 0.531 | 0.577 | 0.500 |
| **RLN** | **< 12** | 0.619 | 0.607 | 0.625 | 0.557 | 0.633 | 0.603 | 0.643 | 0.616 | 0.542 | 0.500 | 0.554 | 0.500 |
|  | **≥12** | 0.638 | 0.596 | 0.637 | 0.580 | 0.635 | 0.583 | 0.648 | 0.573 | 0.606 | 0.552 | 0.608 | 0.500 |

Abbreviations: RLNs: Retrieval lymph nodes; LNR: The ratio of metastatic to retrieved nodes; LODDS: The log odds of lymph node ratio; LONT: The log odds of negative lymph nodes/T stage; AUC: Area under the curve.
